# Are the atrial natriuretic peptides a missing link predicting low‐voltage areas in atrial fibrillation? Introducing the novel biomarker‐based atrial fibrillation substrate prediction (ANP) score

**DOI:** 10.1002/clc.23378

**Published:** 2020-05-27

**Authors:** Timm Seewöster, Petra Büttner, Samira Zeynalova, Gerhard Hindricks, Jelena Kornej

**Affiliations:** ^1^ Department of Electrophysiology Heart Center at Leipzig University Leipzig Germany; ^2^ Institute for Medical Informatics, Statistics, and Epidemiology, Leipzig University Leipzig Germany; ^3^ School of Medicine, Cardiovascular Medicine Boston University Boston Massachusetts USA

**Keywords:** atrial fibrillation, atrial myopathy, biomarkers, electro‐anatomical substrate, NT‐proANP, score

## Abstract

**Background:**

In patients with atrial fibrillation (AF), left atrial (LA) enlargement, and the presence of low‐voltage areas (LVAs) indicate an advanced disease stage. NT‐proANP is a biomarker, which is significantly higher in both phenotypes. Prediction of LVAs before catheter ablation could impact the prognosis and therapeutical management in AF patients.

**Objective:**

The aim of this study was to (a) analyze the predictive value of a novel biomarker‐based AF substrate prediction score, and (b) compare it with DR‐FLASH and APPLE scores.

**Methods:**

Patients undergoing first AF catheter ablation were included. LA volume (LAV) was analyzed prior to ablation using cardiovascular magnetic resonance imaging (CMR). Blood plasma samples from the femoral vein were collected before AF ablation. NT‐proANP was analyzed using commercially available assays. LVAs were determined using high‐density maps during catheter ablation and defined as <0.5 mV. The novel ANP score (one point for **A**ge ≥ 65 years, **N**T‐proANP > 17 ng/mL, and **P**ersistent AF) was calculated at baseline.

**Results:**

The study population included 156 AF patients (64 ± 10 years, 65% males, 61% persistent AF, 28% LVAs). The cut‐off ANP score ≥ 2 demonstrated 77% sensitivity and 70% specificity. On logistic regression (odds ratio [OR] 3.469) and receiver operating characteristic (ROC) analysis (area under the curve [AUC] 0.778, *P* < .001), the ANP score significantly predicted LVAs presence. There were no differences between novel ANP score – which is a new one ‐ is described in the Abstract; with APPLE (AUC 0.718, *P* = .378) and DR‐FLASH (AUC 0.766, *P* = .856) scores.

**Conclusions:**

The novel biomarker‐based ANP score demonstrates good prediction of LVAs.

## 1 INTRODUCTION

Atrial fibrillation (AF) is the most common sustained arrhythmia worldwide and is associated with relevant adverse events, impaired quality of life, intensive healthcare costs, and mortality. Interventional AF ablation is a key treatment for highly symptomatic AF patients.^1^ Several clinical variables, blood biomarkers, and imaging parameters are associated with AF progression. The presence of low‐voltage areas (LVAs) is a surrogate parameter of advanced electro‐anatomical remodeling in the left atrium (LA) and is associated with significantly worse outcomes after pulmonary vein isolation (PVI).^2^ LVAs are present in approximately 20% to 25% of all AF patients and may require additional ablation strategies, continuation of antiarrhythmic drug therapy, intensive follow‐up,^2^ resulting in higher healthcare costs.

Pathophysiological, electrical, and structural atrial remodeling plays an important role in AF pathogenesis,^3^, ^4^ and is associated with endothelial damage, inflammation, and fibrosis.^5^ Blood biomarkers are widely used in epidemiological and clinical studies to improve the clinical assessment of AF.^6^ There are several biomarkers such as galectin, GDF‐15, or GDF‐23, which are markers of pro‐fibrotic changes and may play a role in the prediction for LVAs. However, recent studies analyzing prediction of LVAs using pro‐fibrotic biomarkers are inconclusive.^7^, ^8^, ^9^ Natriuretic peptides (NP) as NT‐proANP, are predominantly released in the atrial myocardial wall as a response to atrial wall stretch.^10^ Underlying diseases, such as hypertension or heart failure, increase atrial stretch leading to LA dilatation, and initiation of AF. Consequently, elevated NP levels were found to be associated with an increased risk of AF initiation, and AF recurrence after intervention.^11^

The LVAs prediction remains challenging and is not widely available.^12^ It requires either special algorithms of the cardiovascular magnetic resonance imaging (CMR)^12^ or invasive electro‐anatomical mapping.^2^ LVAs prediction before PVI would be very helpful, because in the case of high probability of LVAs presence, the interventionalist could choose RF ablation instead of cryoballoon ablation, where only PVI without individualized linear ablation is possible. There are only few clinical scores aimed to predict LVAs in AF patients.^13^, ^14^ While the DR‐FLASH score was introduced to predict LVAs first,^14^ the APPLE score was developed for the prediction of AF recurrences,^13^, ^15^ and later had been also proven as the LVAs prediction score.^16^

Therefore, the aim of this study was to (a) analyze the predictive value of novel biomarker‐based AF substrate prediction score, and (b) compare it with APPLE and DR‐FLASH scores.

## 2 METHODS

In the present study, we included 156 patients who underwent AF catheter ablation between October 2015 and April 2017 at the Heart Center Leipzig (Germany). Inclusion criterion was a highly symptomatic and refractory to antiarrhythmic treatment AF. Exclusion criteria were pregnancy, age <18 or >75, valvular AF, cancer, acute, or systemic inflammatory diseases. The studies were approved by the local Ethical Committee (Medical Faculty, University of Leipzig) and patients provided written informed consent for participation. Paroxysmal and persistent AF were defined according to the current guidelines.^17^ Paroxysmal AF was defined as self‐terminating within 7 days after onset. Persistent AF lasted longer than 7 days or required drugs or direct current cardioversion for termination.

### 2.1 Cardiovascular magnetic resonance imaging

Prior to AF ablation, patients underwent 1.5 T CMR (Ingenia, Philips Medical) 1 to 2 days before the intervention as previously described.^18^ Briefly, a contrast‐enhanced MR angiography of the left atrium and the pulmonary veins was acquired during breath‐holding without electrocardiogram (ECG) gating using real‐time bolus tracking. CMR data were reviewed and total LA volume (LAV) was determined after exclusion of the atrial appendage (LAA) and the pulmonary veins (PV).

### 2.2 Catheter ablation

The electro‐anatomical mapping was performed as previously described.^19^ Briefly, two three‐dimensional (3D) mapping systems (Carto, Biosense Webster, Diamond Bar, California or EnSite Precision, St. Jude Medical [SJM], Saint Paul, Minnesota) were used for electro‐anatomical mapping. The spiral mapping catheter used for NavX Ensite procedures was the Reflexion Spiral (SJM) and the Carto Lasso (Biosense Webster) for Carto3 procedures. In both mapping systems, the cut‐off values for defining LVAs were identical: <0.5 mV for low voltage and >0.5 mV for normal voltage. Patients underwent high‐density mapping of LA voltage using multipolar catheters in combination with auto‐annotation algorithms (AutoMap in Precision and ConfiDense in Carto 3). The voltage mapping points were obtained in sinus rhythm before ablation or after ablation of the pulmonary veins. Here, the number of points was >1000. According to the presence of LVAs individually tailored ablation lines were added after the electro‐anatomical map. Only patients with electro‐anatomical substrate leading to additional linear ablations were considered as the LVAs group.

### 2.3 Blood samples

Blood samples were obtained in ethylenediaminetetraacetic acid (EDTA) test tubes in fasting state prior ablation procedure from the femoral vein and processed within 1 hour of collection. Blood plasma was prepared (1000×*g* for 10 minutes at 20°C) and aliquots were stored at −70°C for subsequent analysis. NT‐proANP levels were studied using Luminex Screening Assay (R&D/bio‐techne, Minneapolis, Minnesota).

### 2.4 Scores

The APPLE score (one point for **A**ge ≥ 65 years, **P**ersistent AF, im**P**aired eGFR<60 mL/min/1.73 m^2^, **L**eft atrial (LA) diameter ≥ 43 mm, **E**F < 50%) and the DR‐FLASH score (one point for **D**iabetes mellitus, **R**enal dysfunction, persistent **F**orm of AF, **L**A diameter > 45 mm, **A**ge > 65 years, female **S**ex, and **H**ypertension), which was developed for the prediction of LVAs, were calculated before catheter ablation using baseline patients' characteristics.^13^, ^14^ The novel ANP score (one point for **A**ge ≥ 65 years, **N**T‐proANP over 75th percentile in peripheral circulation [≥17 ng/mL], and **P**ersistent AF type) was built at baseline prior to ablation. The cut‐off ≥2 had been chosen to facilitate comparison of all scores and enable interpretation of previous and current results.

### 2.5 Statistics

Data are presented as mean and SD for normally distributed or median (interquartile range) for skewed continuous variables, and as proportions for categorical variables. The differences between continuous values were assessed using an unpaired *t*‐test or the Mann‐Whitney, and a χ² test for categorical variables.

Logistic regression analysis was used to identify factors associated with LVAs. Multivariable analysis, which included variables with a *P*‐value <.05 found on univariable analysis, was performed to identify independent predictors for the presence of LVAs.

Receiver operating characteristic (ROC) curves were generated for a graphical illustration of ANP, DR‐FLASH, and APPLE scores' performance in predicting LVAs, with the area under the curve (AUC) being equivalent to the c‐index for determining the predictive value for a score. The c indices (ie, areas under the ROC curves) for the two scores were compared by using DeLong's method.^20^

A *P*‐value <.05 was considered statistically significant, and all analyses were performed with SPSS statistical software version 25 (SPSS Inc., Chicago, Illinois).

## 3 RESULTS

### 3.1 Clinical characteristics of the study population

In total, 156 patients (64 ± 10 years, 65% males, 61% persistent AF, 28% LVAs) undergoing primary AF ablation were included in the study. Clinical characteristics of the study population are summarized in Table 1. Patients with LVAs were significantly older, had more frequently persistent AF, a lower estimated glomerular filtration rate (eGFR) (all *P* < .01), and were more often women (*P* = .041). CMR derived LAV was significantly higher in patients with LVAs (median 68 mL/m^**2**^ (interquartile range (IQR) 55‐79) vs 52 mL/m^**2**^(IQR 43‐67), *P* < .001), and NT‐proANP levels were significantly higher in patients with LVAs (median 14 ng/mL (IQR 8‐21) vs 10 ng/mL (IQR 6‐14), *P* = .003).

**TABLE 1:** Clinical characteristics of the study population

	Total population n = 156	LVAs		*p*‐value
		Yes (n = 44)	No (n = 112)	
Age, years	64 (57‐72)	68 (64‐74)	61 (55‐69)	<.001
Women	55 (35)	21 (48)	34 (30)	.041
Persistent AF	96 (61)	37 (84)	58 (52)	<.001
BMI, kg/m^2^	30 (26‐33)	31 (26‐34)	29 (26‐33)	.168
eGFR, mL/min/1.73 m^2^	79 (66‐89)	70 (62‐82)	81 (69‐93)	.001
LAV, mL/m^2^	56 (46‐73)	68 (55–79)	52 (43–67)	<.001
LV‐EF, %	56 (48‐61)	58 (45‐65)	57 (50‐65)	.267
NT‐proANP, ng/mL	11 (6‐17)	14 (8–21)	10 (6–14)	.003
CHA_2_DS_2_‐VASc score	3 (1–4)	3 (2–4)	2 (1‐4)	<.001
APPLE score	2 (1–3)	3 (2–4)	2 (1‐3)	<.001
DR‐FLASH score	4 (2–5)	5 (4–5)	3 (2–4)	<.001
ANP score	1 (1–2)	2 (2–3)	1 (0–2)	<.001

*Note*: Data presented as n (%) or median (IQR).

Abbreviations: AF, atrial fibrillation; BMI, body mass index; eGFR, estimated glomerular filtration rate; IQR, interquartile range; LAV, left atrial volume; LV‐EF, left ventricular ejection fraction.

In the logistic regression univariable (unadjusted) analysis, age, female gender, persistent AF, LA volume, and NT‐proANP were significantly associated with LVAs (Table 2). In multivariable analysis, advanced age (odds ratio [OR] 2.973, 95% confidence interval [CI] 1.199‐7.373, *P* = .019), persistent AF (OR 3.431, 95% CI 1.296‐9.086, *P* = .013), and higher NT‐proANP levels (OR 2.763, 95% CI 1.123‐6.797 *P* = .027) remained significant predictors for LVAs. These variables were used to build the novel biomarker‐based AF substrate prediction—the ANP score.

**TABLE 2:** LVAs prediction using clinical variables

Variables	Univariable analysis			Multivariable analysis		
	OR	95% CI	*P*‐value	OR	95% CI	*P*‐value
Age ≥ 65 years	3.970	1.850‐8.519	<.001	2.973	1.199‐7.373	.019
Women	2.095	1.024‐4.285	.043	1.971	0.831‐4.673	.124
Persistent AF	4.921	2.023‐11.969	<.001	3.431	1.296‐9.086	.013
eGFR ≤ 60 mL/min	2.430	0.928‐6.366	.071	0.925	0.290‐2.952	.895
LAV ≥ 56 mL/m^2^	4.702	2.115‐10.451	<.001	2.182	0.891‐5.346	.088
NT‐proANP ≥ 17 ng/mL	3.969	1.817‐8.668	.001	2.763	1.123–6.797	.027

Abbreviations: AF, atrial fibrillation; eGFR, estimated glomerular filtration rate; LAV, left atrial volume.

**FIGURE 1:**
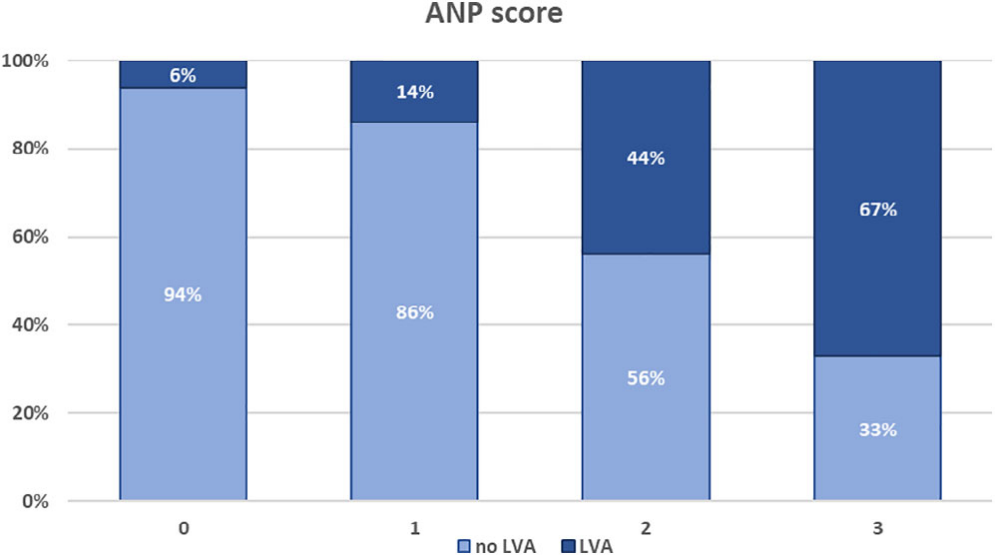
Association between low‐voltage areas (LVAs) presence and the ANP score progression. The figure presents the distribution of LVAs (as percentages) accordingly to the ANP score points: LVAs presence is increased in AF patients with advanced ANP score

There were n = 33 (21%), n = 55 (35%), n = 50 (32%), and n = 19 (12%) patients with ANP scores 0, 1, 2, and 3, respectively. The LVAs presence increased with each ANP score point and was 6%, 14%, 44%, and 67% for ANP scores 0 to 4 **(**Figure 1 **)**.

### 3.2 Scores for LVAs prediction

**FIGURE 2:**
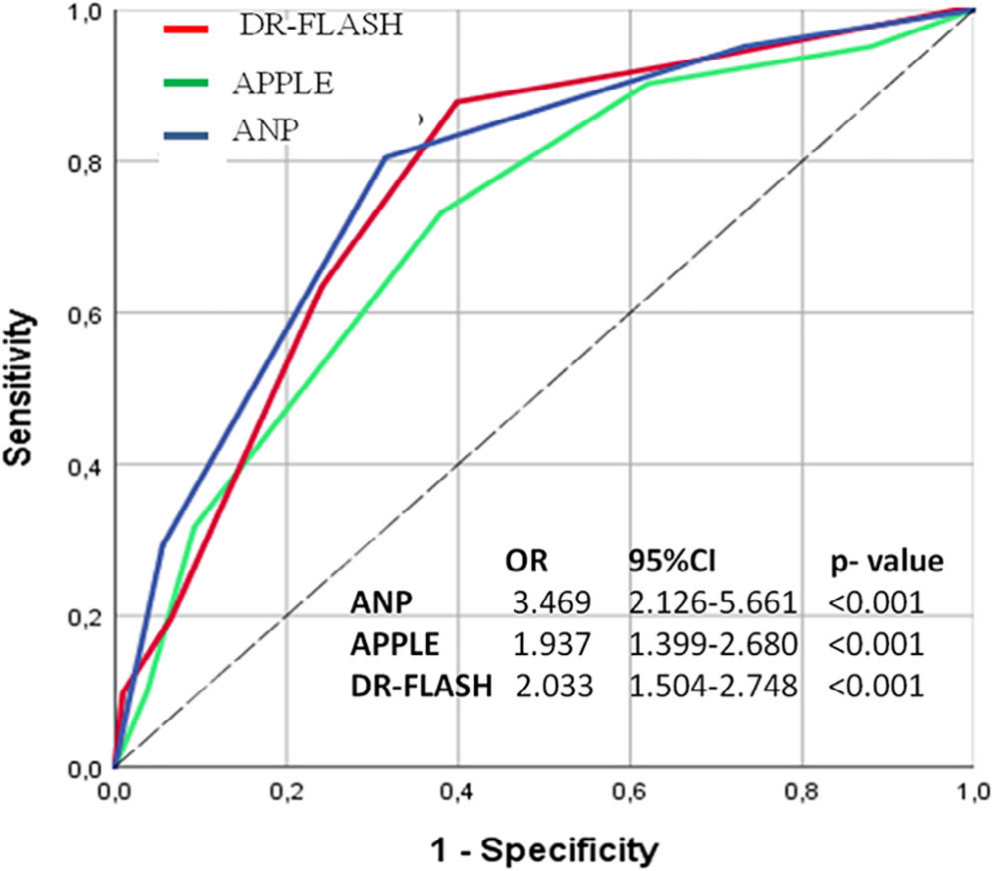
Low‐voltage areas (LVAs) prediction with APPLE, DR‐FLASH and ANP score

All scores were significantly associated with LVAs (Figure 2). The prediction of LVAs in logistic regression was significantly better using ANP score (OR 3.469) than APPLE and DR‐FLASH scores (OR 1.937 and OR 2.033, respectively), while the ROC analyses were similar : AUC 0.778 for the ANP score, AUC 0.718 for the APPLE, and AUC 0.766 for the DR‐FLASH scores (all <.001, Table 3(a)). Comparing the scores using DeLong's method, there were no differences between the scores (*P* = .378 between ANP and APPLE scores, *P* = .856 between ANP and DR‐FLASH scores). The novel ANP score  ≥ 2 demonstrated a good sensitivity and specificity for the LVAs presence (72% and 70%, respectively). The sensitivity/specificity for APPLE and DR‐FLASH scores >2 were 73/63% and 93/65%, respectively.

**TABLE 3:** LVAs prediction using scores (a) in the whole study population (n = 156) and (b) in patients with persistent AF (n = 96)

Variables	Logistic regression			ROC analysis		
	OR	95% CI	*P*‐value	AUC	95% CI	*P*‐value
(a) LVAs prediction using scores in the whole study population (n = 156)						
ANP score	3.469	2.126‐5.661	<.001	0.778	0.696‐0.861	<.001
DR‐FLASH score	2.033	1.504‐2.748	<.001	0.766	0.684‐0.848	<.001
APPLE score	1.937	1.399‐2.680	<.001	0.718	0.627‐0.809	<.001
(b) LVAs prediction using scores in patients with persistent AF (n = 96)						
ANP score	3.396	1.728‐6.674	<.001	0.713	0.608‐0.818	<.001
DR‐FLASH score	2.042	1.353‐3.081	.001	0.717	0.613‐0.822	.001
APPLE score	1.775	1.113‐2.832	.016	0.656	0.543‐0.770	.012

Abbreviations: AF, atrial fibrillation; AUC, area under the curve; CI, confidence interval; LVA, low‐voltage areas; OR, odds ratio; ROC, receiver operating characteristic.

The predictive value of the scores was similar in the subgroup of patients with persistent AF (Table 3(b)).

## 4 DISCUSSION

To the best of our knowledge, this is the first study demonstrating the predictive value of the novel biomarker‐based score for prediction of electro‐anatomical remodeling (LVAs) prior to catheter ablation. We found that LVAs prediction using the novel ANP score was non‐inferior or even better than the DR‐FLASH and APPLE scores. Compared to other scores, the ANP score demonstrated better specificity. Importantly, using only three variables mirroring atrial myopathy with its structural changes, it was possible to build an LVAs score with a good predictive value.

### 4.1 Prediction of electro‐anatomical substrate using clinical variables

The presence of LVAs is a key characteristic of AF progression and pathogenesis. Usually, LVAs are determined invasively using LA voltage mapping.^2^, ^21^ Voltage‐guided substrate modification by targeting LVAs in addition to PVI has been shown to be superior to conventional PVI “only” approaches regarding freedom from AF recurrences after ablation.^12^ However, using other treatment strategies (eg, antiarrhythmic drugs, electrical cardioversion, or even ablation without available assessment of LVAs), it would be helpful to predict electro‐anatomical remodeling already at baseline and prioritize individual treatment opportunities. Hence, the novel biomarker‐based LVAs scores can be used for patients' screening already in the outpatient setting and help choosing the adequate treatment.

A recent study reported that age and persistent AF are clinical significant predictors associated with LVAs.^22^ This is in line with the present study, where persistent AF was associated with an almost 3.5‐fold risk for the presence of LVAs. Of note, similar findings were reported from autopsy studies, where a higher percentage of fibro‐fatty infiltration was found in patients with persistent AF.^23^ In our study, besides persistent AF, age ≥ 65 years proved to be a powerful predictor in multivariable analysis and was associated with an almost 3‐fold risk for LVAs occurrence. While recent pathologic findings do not support a solely age‐related increase in fibrosis,^23^ several studies demonstrated a strong association between age, AF occurrence,^24^ and LVAs presence.^25^ Furthermore, current AF management guidelines list “age‐related fibrosis” as an etiological factor underlying AF.^17^

Renal dysfunction is another controversial clinical factor associated with LVAs. Compared to other observations, where impaired renal function was associated with atrial fibrosis,^26^ we did not find this association in our current analysis. This could be explained by only modest eGFR reduction in the LVAs group compared to the non‐LVAs group. Also, our study cohort was relatively young and healthy, and only few patients had renal impairment accordingly to stage III, while none of our patients had stages IV (pre‐dialysis) and V (dialysis).

LA enlargement had been used as a marker of advanced electro‐anatomical remodeling because of its strong association with AF progression and related success of different AF management strategies.^27^, ^28^, ^29^ The DECAAF study demonstrated atrial fibrosis detection using delayed enhancement CMR.^12^ However, beside high‐volume tertiary centers, CMR is not available for many EP labs worldwide. Recently, we demonstrated the importance of CMR measurements assessing different LA parameters and their association with LVAs.^18^ We found that CMR derived LA volume was superior to different monoplane LA diameters. In addition to “passive” LAV measurements, we could also show that the “active” LA function was associated with LVAs.^25^ However, in the present study, the association between LAV and LVAs was not significant.

### 4.2 Blood biomarkers and electro‐anatomical remodeling

Besides NT‐proANP in our current analysis and in the previously published study by Yin et al,^30^ no other biomarkers were included into clinical scores for the prediction of the LVAs. While histological analyses of atrial appendages suggested an association between interstitial fibrosis and galectin‐3 (Gal‐3) in patients undergoing cardiac surgery,^8^ the study by Begg et al could not demonstrate any association neither between LVAs nor recurrences and pro‐fibrotic biomarkers (such as Gal‐3, type III procollagen N‐terminal peptide, fibroblast growth factor 23, or type I collagen C‐terminal telopeptide).^7^ These findings are in accordance with previous results demonstrating that modification of CHADS_2_ and CHA_2_DS_2_‐VASc scores by adding TGF‐ß1 did not improve the prediction of arrhythmia recurrences.^31^

In current study, NT‐proANP **≥**17 ng/mL was associated with almost 3‐fold risk for the LVAs presence. This finding is in line with a previous study, which proved an association of NT‐proANP levels with LVAs and persistent AF.^19^ Because of its secretion from the atria as a response to atrial stress, NT‐proANP might be considered as a surrogate parameter for atrial myopathy. However, although recent epidemiological studies found that among natriuretic peptides, NT‐proBNP was strongly associated with incident AF,^32^, ^33^ the results were inconsistent.^34^, ^35^ Smith et al^34^ reported that among natriuretic peptides, NT‐proANP showed better prediction of incident AF in a general population compared to NT‐proBNP (OR 1.67 vs 1.45, respectively). Our recent study partly confirmed these results demonstrating that NT‐proANP, and not NT‐proBNP levels, were significantly associated with AF progression phenotypes in clinical cohort.^19^ Therefore, our previous and current results indicate that NT‐proANP could be a missing link in LVAs prediction in AF patients.

### 4.3 Prediction of electro‐anatomical substrate using scores

There is a vast majority of different scores for the prediction of AF incidence, thromboembolic complications, AF recurrences, or mortality associated with AF.^36^ However, there are only a few scores that were designed to predict LVAs.

Besides APPLE and DR‐FLASH scores, the MB‐LATER score (one point for **M**ale sex, **B**undle brunch block, **L**eft **A**trium ≥ 47 mm, clinical **T**ype of AF, and **E**arly **R**ecurrent AF) has been tested for LVAs prediction.^37^ In this comparison, the APPLE (OR 1.789, *P* < .001) and the DR‐FLASH score (OR 2.144, *P* < .001) showed a good prediction for LVAs, whereas the MB‐LATER score did not show a significant association with LVAs. Also, in a small analysis using the CHADS_2_ score (one point for **C**ongestive heart failure, **H**ypertension, **A**ge ≥ 75, **D**iabetes mellitus, and two points for previous **S**troke), the cutoff ≥3 was found to be associated with a decreased left atrial voltage.^38^

In the present study, we found that the novel ANP score was associated with LVAs and could be used for their prediction prior catheter ablation. The ANP score  ≥ 2 demonstrated 77% sensitivity and 70% specificity, while the sensitivity and specificity for DR‐FLASH and APPLE scores were 93/65% and 73/63%, respectively. Also, ANP score demonstrated best prediction value on logistic regression and comparable AUC at the ROC analysis—not only in the whole population, but also in a subgroup with persistent AF only. While the prediction of electro‐anatomical substrate using DR‐FLASH score showed the highest sensitivity compared to the other scores, the novel ANP score had a better specificity. Importantly, DR‐FLASH includes mainly clinical factors per se associated with AF, for example, hypertension, diabetes, renal dysfunction—which are most important AF bystanders. In contrast, the ANP score included only variables, which pathophysiological are very specific to describe atrial myopathy and are associated with AF initiation and progression. The only limiting factor might be NT‐proANP measurement, as it is not widely available, yet. However, our previous and current results demonstrate that NT‐proANP seems to be a specific blood marker of “stressed” atria,^19^, ^34^ paving the way toward larger studies proving this hypothesis.

### 4.4 Strengths and limitations

The strength of the present study is a deeply phenotyped AF cohort with available clinical (age, sex, persistent AF, BMI), imaging (echocardiography, CMR), peri‐interventional (electro‐anatomical mapping) data, and blood samples of patients undergoing AF ablation. Despite the advantage of such unique phenotyping, this could be considered as a disadvantage, because validation of our results in an external cohort is problematic—not all ablation centers analyze electro‐anatomical mapping or have available blood biobanking. Therefore, missing validation of the novel score is the major limitation of the present study.

Also, since quantitative measurements (eg, LVAs area or indexed LVAs area) were not performed in this cohort, a correlation analyses between LVAs areas' size and NT‐proANP concentration was not possible.

Another limitation is not consistently obtained follow‐up data as many patients were followed by general practitioners or cardiologists in the outpatient setting. Also, compared to our previous studies, there were significant differences in follow‐up management among those patients, who presented to our clinic after 2014.^37^ However, rhythm outcomes follow‐up is of clinical interest and should be addressed in future randomized trials.

## 5 CONCLUSION

The novel biomarker‐based ANP score demonstrated good prediction of LVAs. Compared to other established AF substrate scores, the ANP score includes parameters associated with atrial myopathy and demonstrates better specificity.

## CONFLICT OF INTEREST

The authors declare no potential conflict of interests.
